# Magnet anode enhances extracellular electron transfer and enrichment of exoelectrogenic bacteria in bioelectrochemical systems

**DOI:** 10.1186/s13068-019-1477-9

**Published:** 2019-05-31

**Authors:** Huihui Zhou, Xiaoxue Mei, Bingfeng Liu, Guojun Xie, Defeng Xing

**Affiliations:** State Key Laboratory of Urban Water Resources and Environment, School of Environment, Harbin Institute of Technology, P.O. Box 2614, 73 Huanghe Road, Nangang District, Harbin, 150090 Heilongjiang China

**Keywords:** Microbial fuel cell, Static magnetic field, Magnet anode, Microbial community

## Abstract

**Background:**

Optimizing the ability of exoelectrogens is a key factor in boosting the overall efficiency of bioelectrochemical systems. In this study, we construct magnetic microbial fuel cells (MFCs) with magnets with different static magnetic field (SMF) intensities for use as anodes. It is proposed as an in situ study of the effects of magnetic fields on the performance and exoelectrogenic biofilm of bioelectrochemical system.

**Results:**

The magnetic MFCs obtain a 71.0–105% increase in voltage production and a 42.9–104% increase in power density compared with non-magnetic MFCs. MFCs with a MF intensity of 80 mT obtain the best performances. SMF decreases the internal resistance of MFCs, especially its diffusion resistance. The relative abundance of *Geobacter* in magnetic MFCs is up to 32.5% higher than that of non-magnetic MFC. SMFs also lead to the shifts in microbial community structure of methanogens.

**Conclusion:**

The constructed magnetic MFCs obtained better performance compared with the non-magnetic MFC, in terms of voltage production, power density, and coulombic efficiency. The relative abundance of *Geobacter* spp. (one kind of exoelectrogen) was much higher in the magnetic MFCs. The optimal static magnetic field intensity for enriching exoelectrogens is around 80 mT. It is likely that the decrease of internal resistance, enrichment in exoelectrogens, and the syntrophic interactions between exoelectrogens and methanogens result in the enhanced performance of magnetic MFCs. This study provides a magnetic method for the enrichment of exoelectrogens, which can be extensively applied in bioelectrochemical systems. 
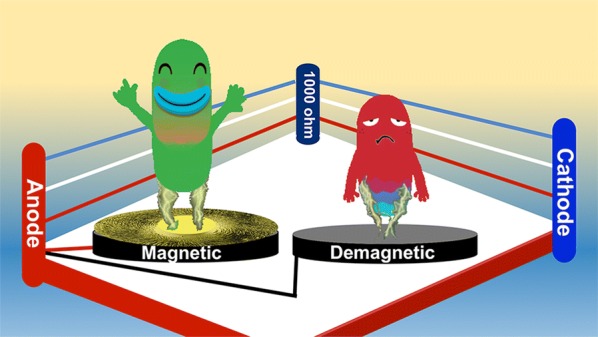

## Background

Bioelectrochemical systems (BESs) are a promising wastewater treatment approach that combines waste remediation with energy generation, in which the anode microorganisms oxide biodegradable substrates to generate current [1]. The application of BESs has expanded from electricity production (microbial fuel cells) to hydrogen generation (microbial electrolysis cells), compounds synthesis (microbial electrosynthesis), promote desalination (microbial desalination cell), or remediation of pollutants (microbial remediation cells) [2]. The different kinds of BESs share one common principle, in that the semi-cell reaction of the anode relies on exoelectrogens [3]. Optimizing the ability of anodic exoelectrogens is thus a key factor in boosting the overall efficiency of BESs.

The most extensively studied exoelectrogens used in BESs belong to *Geobacter* spp. and *Shewanella* spp., and these are capable of transferring electrons to an electrode through c-type cytochromes [4], conductive pili [5, 6], or electron shuttles [7]. In subsequent studies, an increasing number of exoelectrogens have been identified, and these are mainly distributed in *Proteobacteria*, including *Alpha*-, *Beta*-, *Gamma*-, *Delta*-, and *Epsilon*-*proteobacteria* [8]. In addition, microorganisms among the phyla *Firmicutes* [9], *Acidobacteria* [10], and *Actinobacteria* [11] have also demonstrated electrochemical activity. It is generally believed that the electricity production capacity of pure cultured strains is smaller than that of mixed strains, with the exception of *Geobacter* *sulfurreducens* PCA [12] and *Rhodopseudomonas palustris* DX-1 [13]. As such, it is likely that directing the microbial community structure by optimizing anode materials [14] and environmental parameters (such as pH, temperature, and inoculum) could enhance the performance of BESs [15–17]. When the dominant population in the community belongs to *G. sulfurreducens*, the system generally produces high power density and coulombic efficiency [18]. In view of this, specific enrichment of *Geobacter* spp. should contribute to the further application of BESs.

Magnetic fields (MFs) have been used in BESs and have been suggested to promote the power generation of BESs [19]. It has been speculated that the enhancement of MFCs performance via a MF is likely due to the oxidative stress and magnetohydrodynamic effects [20]. Previous studies showed that MF can affect the growth and biodegradation ability of bacteria [21, 22]. A weak MF leads to substrate removal and microbial growth with an increase rate up to 44% [23]. Bacterial diversity was decreased under magnetic-exposed condition [24]. Magnetic field also facilitates the cytochrome c-mediated bioelectrochemical transformations rates and enhances performance of biofuel cells with different enzyme assemblies attached on electrodes [25, 26]. In our previous study, we found that pulse electromagnetic fields can enhance extracellular electron transfer (EET) and facilitated exoelectrogens (*Geobacter*) enrichment on the anode surface of MFCs resulting in high power generation [27]. The growth of microorganisms can be stimulated or inhibited depending on the intensity of a MF [28], and the appropriate range of a MF intensity that would serve to enrich exoelectrogens, thus enhancing the power generation of MFCs, has not yet been elucidated. As such, the use of MFs in BESs still needs to be explored further. Additionally, the direction and intensity of a pulsed magnetic field are constantly changing, which can affect the aggregation of microorganisms and, in turn, affect the stability of exoelectrogens. In contrast, a static magnetic field (SMF) provided by a magnet is stable in both direction and intensity, which could be conducive to the stability of the microorganisms present in BESs. Magnets have good conductivity and can be constructed at different sizes and thicknesses according to the application requirements. To explore the effects of magnetic fields on MFC performance and the formation of exoelectrogens directly, we constructed MFCs utilizing magnets as anodes. The magnets of different SMF intensities were obtained utilizing different thermal-demagnetizing temperatures. The response of exoelectrogenic communities to different intensities of SMFs in MFCs was analyzed based on Illumina HiSeq sequencing of 16S rRNA gene amplicons.

## Methods

### MFC configuration and operation

**Fig. 1:**
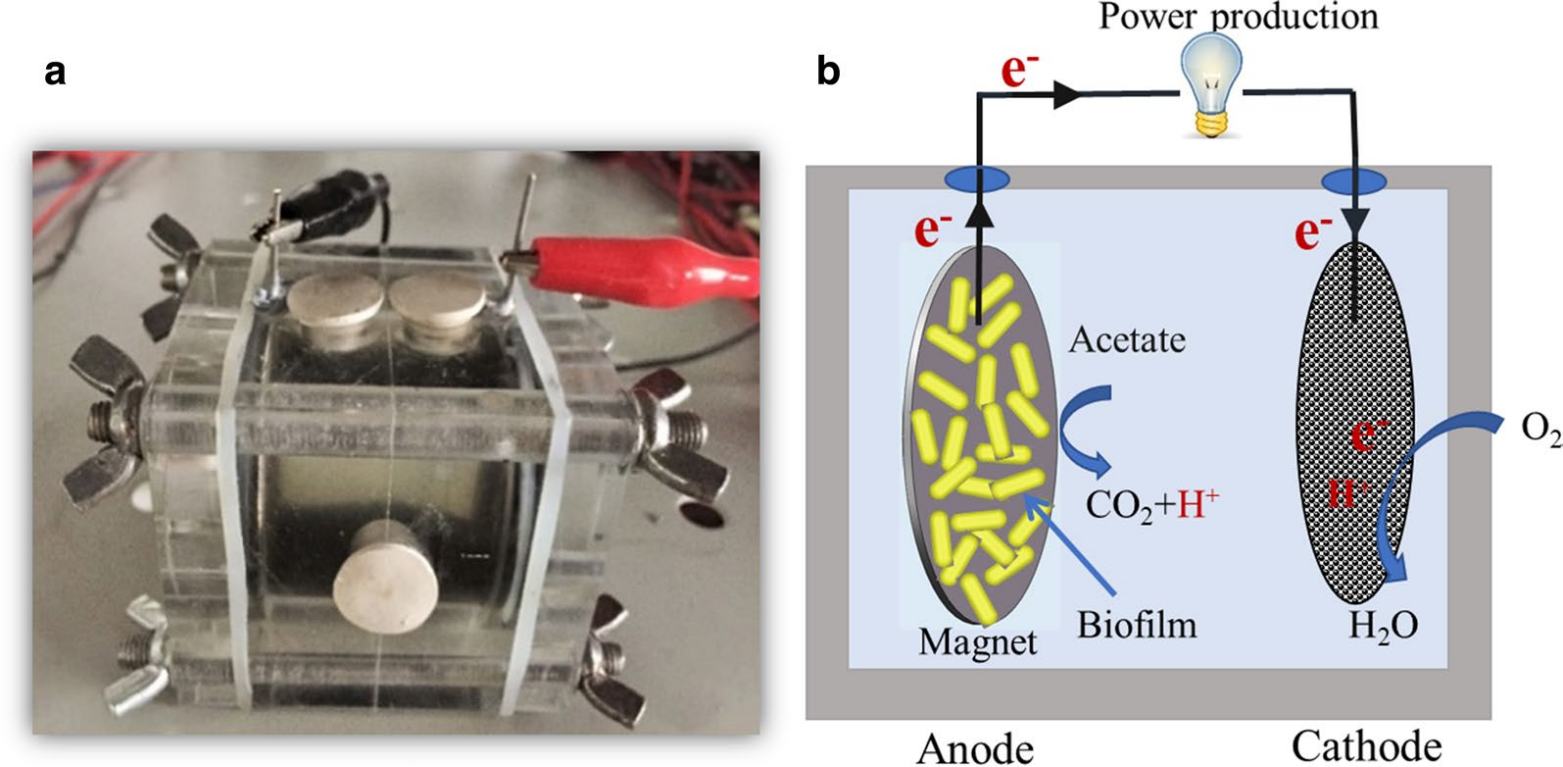
MFC reactor prototype (**a**) and schematic diagram (**b**)

The constructed single-chamber MFC reactor was cubic and the chamber inside was cylindrical with a valid volume of 28 mL (Fig. 1). The anodes were cylindrical neodymium iron boron (NdFeB) magnets measuring 35 mm in diameter and 3 mm in thickness (Buda Metal Products Co., Ltd., Shanghai, China). MFC-0 mT, MFC-20 mT, MFC-80 mT, and MFC-160 mT represent the MFCs with magnet anodes of SMF intensities of 0 mT, 20 mT, 80 mT, and 160 mT, respectively. The magnets were demagnetized at 400 °C, 200 °C, and 100 °C for 30 min in an N_2_ atmosphere to obtain magnets with MF intensity of 0, 20 mT, and 80 mT, respectively. The magnets without thermal-demagnetizing had an initial MF intensity of 160 mT. MF intensity was measured by using a Gausser (WT-10A; TES Electrical Electronic Corp, China). The cathodes were the rolling activated carbon and polytetrafluoroethylene (PTFE) air–cathodes [29]. Both the anodes and cathodes had projected surface areas of 7 cm^2^, and titanium wires were used to connect them across an external resistor of 1000 Ω.

The culture medium of the reactors contained: 2 g/L of sodium acetate, 50 mM of phosphate buffer solution (PBS), 10 mL/L of mineral solution, and 10 mL/L of vitamin solution [27]. The inoculum of the reactors consisted of the activated sludge taken from the secondary clarifier of a local wastewater treatment plant (Harbin, China). The inoculum was added into the reactor by mixing with the culture medium at a volume ratio of 1:5. When the output voltage of the reactor was lower than 50 mV, the medium was replaced with a fresh one. The reactors were operated at a constant temperature room (35 ± 2 °C).

### Electrochemistry analysis

The current generation of the MFCs was obtained by recording the voltage passing through an external circuit resistor (1000 Ω). The voltage was recorded automatically every 10 min by using a multichannel data collector (Model 2700; Keithley Instruments Inc., USA). Electrochemical impedance spectroscopy (EIS) and linear sweep voltammetry (LSV) measurements were conducted on an Autolab Potentiostat/Galvanostat (Autolab PGSTAT 128N, Metrohm Autolab Inc., the Netherlands). During the tests, the anode of the MFC was used as the working electrode, and the cathode was used as the counter and the reference electrodes [30]. The scan voltage used in the LSV tests was started from an open circuit voltage (OCV) to 0 V, and the scan rate was 0.1 mV/s. Polarization and power density curves were obtained according to the LSV results. EIS tests of the MFCs were conducted at an OCV condition. The frequency range was from 100 kHz to 10 mHz. The Nyquist plots were analyzed by fitting to an equivalent circuit of *R*_s_ (*R*_ct1_*Q*_1_) (*R*_ct2_*Q*_2_) in ZSimpWin 3.10 (Echem Software). *R*_s_, *R*_ct1_, and *R*_ct2_ represent the ohmic resistance, charge transfer resistance, and finite diffusion resistance, respectively [31]. The chemical oxygen demand (COD) was analyzed by using method 5220 (HACH Company, Loveland, CO). Coulombic efficiency (*C*_E_) was calculated as follows:

$$C_{\text{E}} = \frac{{8\mathop \smallint \nolimits_{0}^{t} I {\text{d}}t}}{FVM},$$

where *I* is the current (A); *t* is the time (s); *F* is Faraday’s constant (96,485 C/mol/e^−^); *V* is the liquid volume (L); and *M* is the consumed acetate (mol/L) [32].

### Illumina sequencing of 16S rRNA gene amplicons

When the experiment concluded, the magnet anodes were cut into pieces, respectively. The biofilm on the surface of the magnets was extracted, amplified, and purity tested as reported previously [26]. Sequencing libraries were constructed using a TruSeq^®^ DNA PCR-Free Sample Preparation Kit (Illumina, USA). A qualified library was sequenced using an Illumina HiSeq 2500 platform.

Paired-end reads were spliced into raw reads using FLASH (V1.2.7) after removing barcode and primer sequences. Raw reads were filtered using QIIME software (http://qiime.org), and chimeric sequences were removed to acquire effective reads. Sequences with over 97% similarity were assigned to an operational taxonomic units (OTU) by using Uparse software (Uparse v7.0.1001) [33]. The sequence with the highest frequency was regarded as the representative sequence for each OTU and classified to taxonomic identification at a confident threshold of 0.8 using Ribosomal Database Project (RDP) Classifier [34]. Alpha diversity indices were obtained using QIIME software (Version 1.7.0), and non-metric multi-dimensional scaling (NMDS) was conducted using R software (Version 2.15.3).

## Results and discussion

### Effects of the magnetic field on MFC performance

**Fig. 2:**
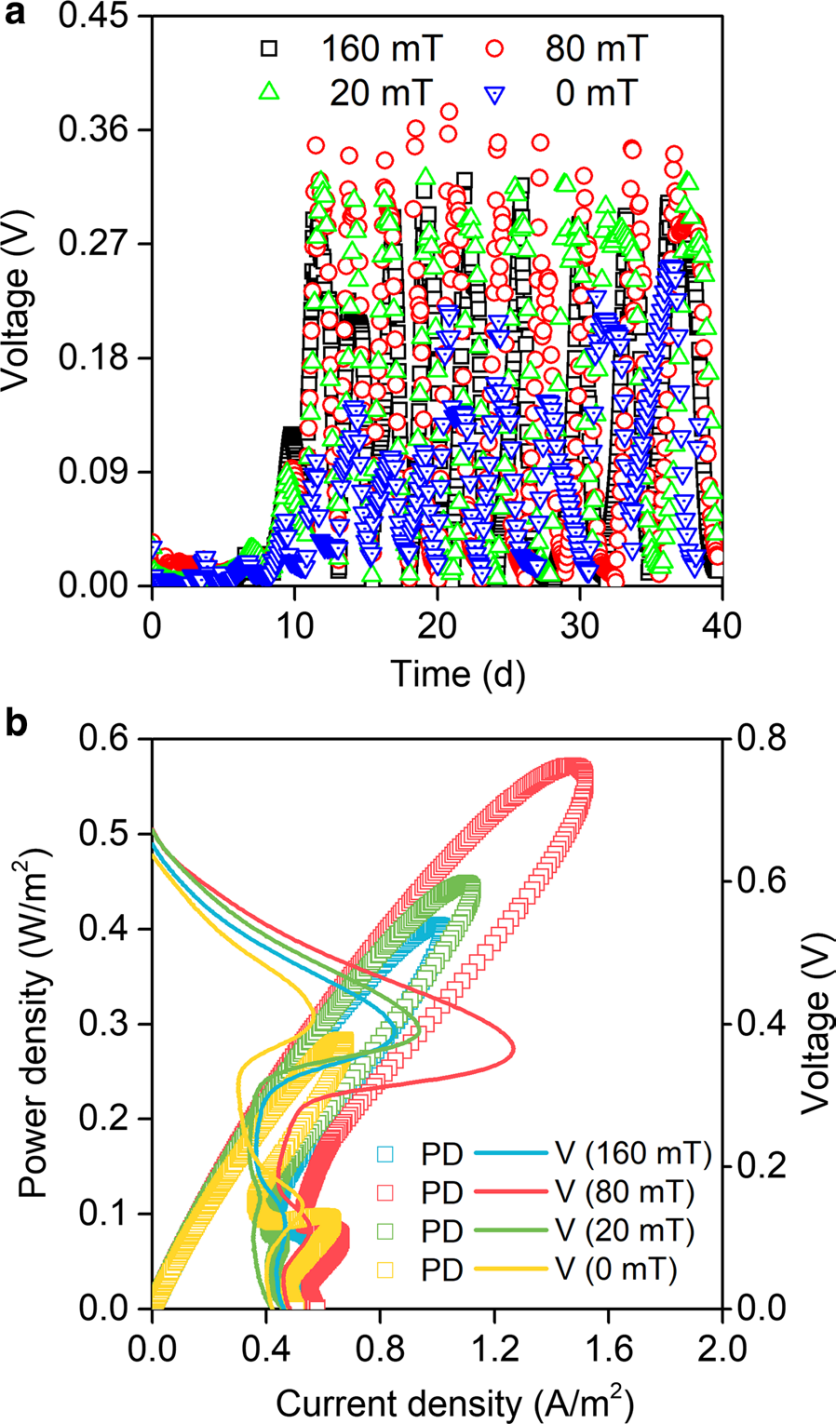
Voltage production (**a**), polarization curves, and power density of MFCs (**b**) with magnets of 0 mT, 20 mT, 80 mT, and 160 mT as anodes. The scatter plots represent power density (PD) and lines represent voltage curves (V)

MFCs with the magnets as the anodes all started up after 9 days of operation (Fig. 2a). The output voltages of the magnetic MFCs (160 mT, 80 mT, and 20 mT) were much higher than that of the non-magnetic MFC (0 mT). The average peak voltage of MFC-80 mT was the highest (350 mV), followed by MFC-20 mT (315 mV), MFC-160 mT (293 mV), and MFC-0 mT (171 mV).

Power density and polarization experiments were conducted on day 23 when the current generation of the MFCs became relatively stable (Fig. 2b). The power density obtained from MFC-160 mT, MFC-80 mT, MFC-20 mT, and MFC-0 mT were 0.4 W/m^2^, 0.57 W/m^2^, 0.45 W/m^2^, and 0.28 W/m^2^, respectively. The power densities generated by the magnetic MFCs were significantly greater than the non-magnetic MFC, with the power density of MFC-80 mT measured around two times higher than that of MFC-0 mT. These results demonstrated that the SMF promoted the power generation of the MFCs, which was also reported in the earlier articles detailing the effect of static magnetic fields or pulse magnetic fields on MFCs [19, 27]. Importantly, the intensity of the SMF needs to be controlled within an appropriate range to obtain the best promotion effect. In this work, we found that a MFC with a magnet anode of 80 mT SMF obtained the best performances.

According to the polarization curves, we can see that all of the reactors showed a similar open circuit potential (OCP) (Fig. 2b). The potential of the MFCs showed small differences in the low current density region, and the differences increased along with the increase of the current density, suggesting that a MF has less effect on the ohmic resistance and the activation resistance, but a greater effect on the diffusion resistance of MFCs. The internal resistance of the MFCs was calculated according to Ohm’s law. Results showed that magnetic MFCs had a much lower internal resistance than non-magnetic MFCs (Table 1), indicating that the SMF could reduce the internal resistance of the MFCs.

**Table 1:** Comparison of performance of MFCs

MFC	Open circuit voltage (V)	Maximum power density (W/m^2^)	Internal resistance (Ω)
160 mT	0.65 ± 0.007	0.40 ± 0.015	586.9 ± 10.5
80 mT	0.67 ± 0.014	0.57 ± 0.008	373.1 ± 7.2
20 mT	0.67 ± 0.012	0.45 ± 0.010	525.3 ± 8.6
0 mT	0.64 ± 0.007	0.28 ± 0.006	912.5 ± 24.2

**Fig. 3:**
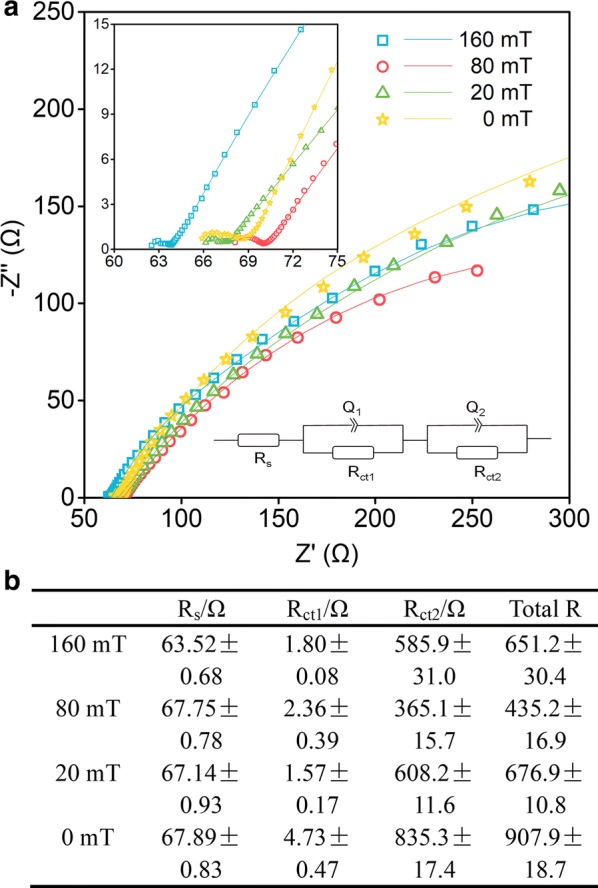
Nyquist plots of MFCs (**a**), the inset is the enlarged curves of the low current density region. The internal resistances of MFCs (**b**) analyzed by fitting to the equivalent circuit: *R*_s_ (*R*_ct1_*Q*_1_) (*R*_ct2_*Q*_2_), where *R*_s_, *R*_ct1_, and *R*_ct2_ represent ohmic resistance, charge transfer resistance, and finite diffusion resistance, respectively

The internal resistances were further analyzed by using electrochemical impedance spectroscopy (EIS) (Fig. 3). According to the EIS analysis, the estimated total resistance of MFC-160 mT, MFC-80 mT, MFC-20 mT, and MFC-0 mT was around 651.2 Ω, 435.2 Ω, 676.9 Ω, and 907.9 Ω, respectively. These results indicated that magnetic MFCs had a much lower internal resistance than non-magnetic MFC, which was consistent with the results calculated by Ohm’s law. It was found that the MFCs all had a similar ohmic resistance, but a lower activation resistance and diffusion resistance was present in the magnetic MFCs. The values for ohmic resistance and activation resistance were relatively small compared to the diffusion resistance, suggesting that transport limitation played a key role in the performance of MFCs with a magnet as the anode. The magnetic MFCs had a much lower diffusion resistance: around 585.9 Ω for MFC-160 mT, 365.1 Ω for MFC-80 mT, and 608.2 Ω for MFC-20 mT, compared with non-magnetic MFC-0 mT (835.3 Ω). Thus, the SMF decreased the internal resistance of the MFCs with magnets as anodes mainly by reducing their diffusion resistance.

**Fig. 4:**
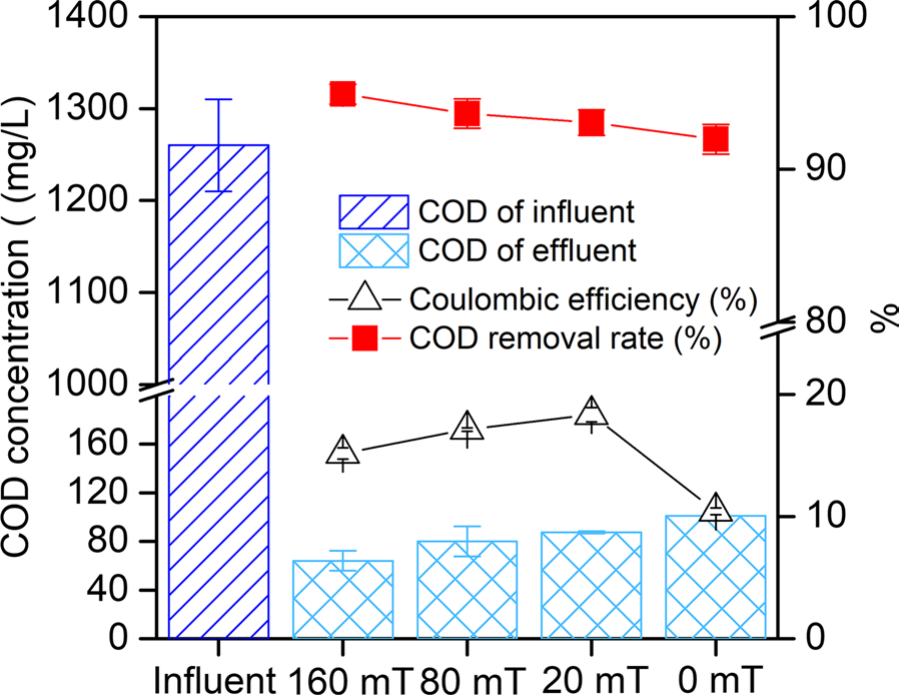
Chemical oxygen demand (COD) of influent and effluent, COD removal rate and coulombic efficiency (CE) of the MFCs

The COD of the influent was around 1260 mg/L, and the final COD values of MFC-160 mT, MFC-80 mT, MFC-20 mT, and MFC-0 mT were around 64 mg/L, 80 mg/L, 87.4 mg/L, and 101.2 mg/L, respectively (Fig. 4). The COD removal rate increased with the increasing intensity of the magnetic field, indicating that the magnetic field promoted the degradation of the substrate, which has also been observed in other wastewater treatment systems [35, 36]. In Fig. 4, we can see that the coulombic efficiency values were around 15.17%, 17.12%, 18.38, and 10.43% in MFC-160 mT, MFC-80 mT, MFC-20 mT, and MFC-0 mT, respectively. The magnetic MFCs obtained higher coulombic efficiencies than the non-magnetic MFCs, suggesting exoelectrogens are more efficient in capturing electron from substrate to produce electricity when exposed to magnetic field. On the other hand, with the increasing of MF intensity, the CE is decreasing, while COD removal rate is increasing, suggesting higher MF intensity does not improve the conversion efficiency of substrate to electricity. In this study, we found that the higher COD removal rates were obtained under higher SMF intensity conditions, while the highest power generation was not obtained in the MFC with the highest SMF intensity (Fig. 4). The results discussed above indicated that different magnetic field intensities generated a mixed effect on MFC performance. Additionally, different bacteria may have different magnetic field tolerances [37]. Moreover, a higher SMF intensity may contribute to higher magnetohydrodynamic effects, increased activity of substrate-degradation bacteria, and inhibit the activity of exoelectrogens.

### Effects of the magnetic field on bacterial community structures of anode biofilms

**Fig. 5:**
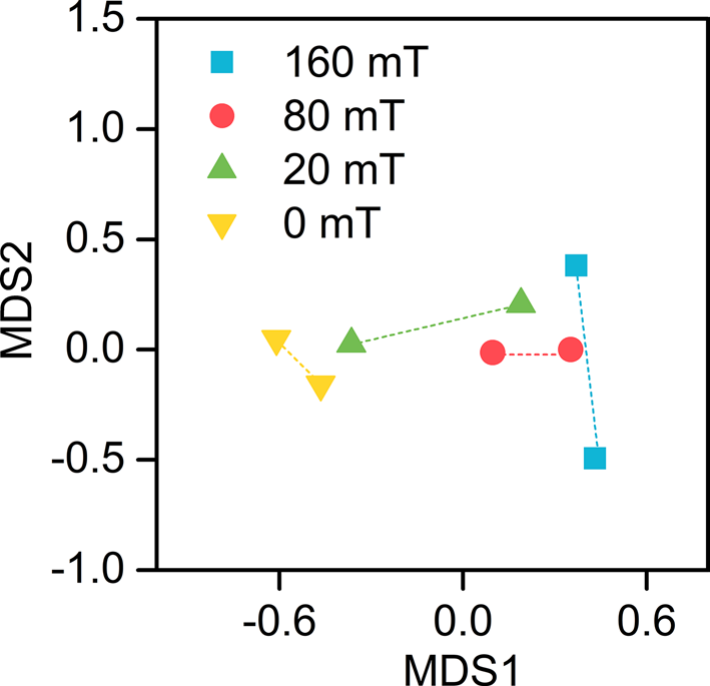
Non-metric multi-dimensional scaling (NMDS) based on bacterial operational taxonomic units (OTUs) of the anode biofilms of MFCs

Results of Illumina HiSeq sequencing displayed that effective reads of each sample ranged from 73,861 to 87,107 (Table 2). Good’s coverage estimator of 0.999 demonstrated that nearly the whole microbial community for each anode biofilm was tracked, which ensured the accuracy of the data. The number of observed operational taxonomic units (OTUs) were 266, 247, 313, and 420 for MFC-160 mT, MFC-80 mT, MFC-20 mT, and MFC-0 mT, respectively, at a threshold of 97%. The values of species richness index (OTUs, Chao1, and ACE) were higher in the non-magnetic MFC; however, values of evenness index (Shannon and Simpson) were similar between the magnetic and non-magnetic MFCs, indicating the SMF reduced the species richness, and has little effect on species evenness. Non-metric multi-dimensional scaling (NMDS) revealed that the bacterial communities of the magnetic MFCs were relatively concentrated (Fig. 5), and they were distinctly separated between magnetic MFCs and non-magnetic MFCs, demonstrating that the SMF led to a shift in the microbial community structure.

**Table 2:** Similarity-based OTUs and species richness and diversity indices at a threshold of 97%

Sample	Effective reads	OTU	Shannon	Simpson (1 − *D*)	Chao1	ACE	Good’s coverage
160 mT	80,001 ± 1049	266 ± 11.6	2.512 ± 0.12	0.609 ± 0.02	271.70 ± 10.1	273.86 ± 4.8	0.999 ± 0
80 mT	86,468 ± 2156	247 ± 9.2	2.397 ± 0.21	0.609 ± 0.03	244.79 ± 12.5	244.44 ± 13.0	0.999 ± 0
20 mT	73,861 ± 1373	313 ± 19.1	2.512 ± 0.13	0.590 ± 0.03	352.14 ± 20.8	361.16 ± 21.2	0.999 ± 0
0 mT	87,107 ± 48	420 ± 14.7	3.056 ± 0.23	0.686 ± 0.06	423.99 ± 16.3	424.44 ± 16.5	0.999 ± 0

**Fig. 6:**
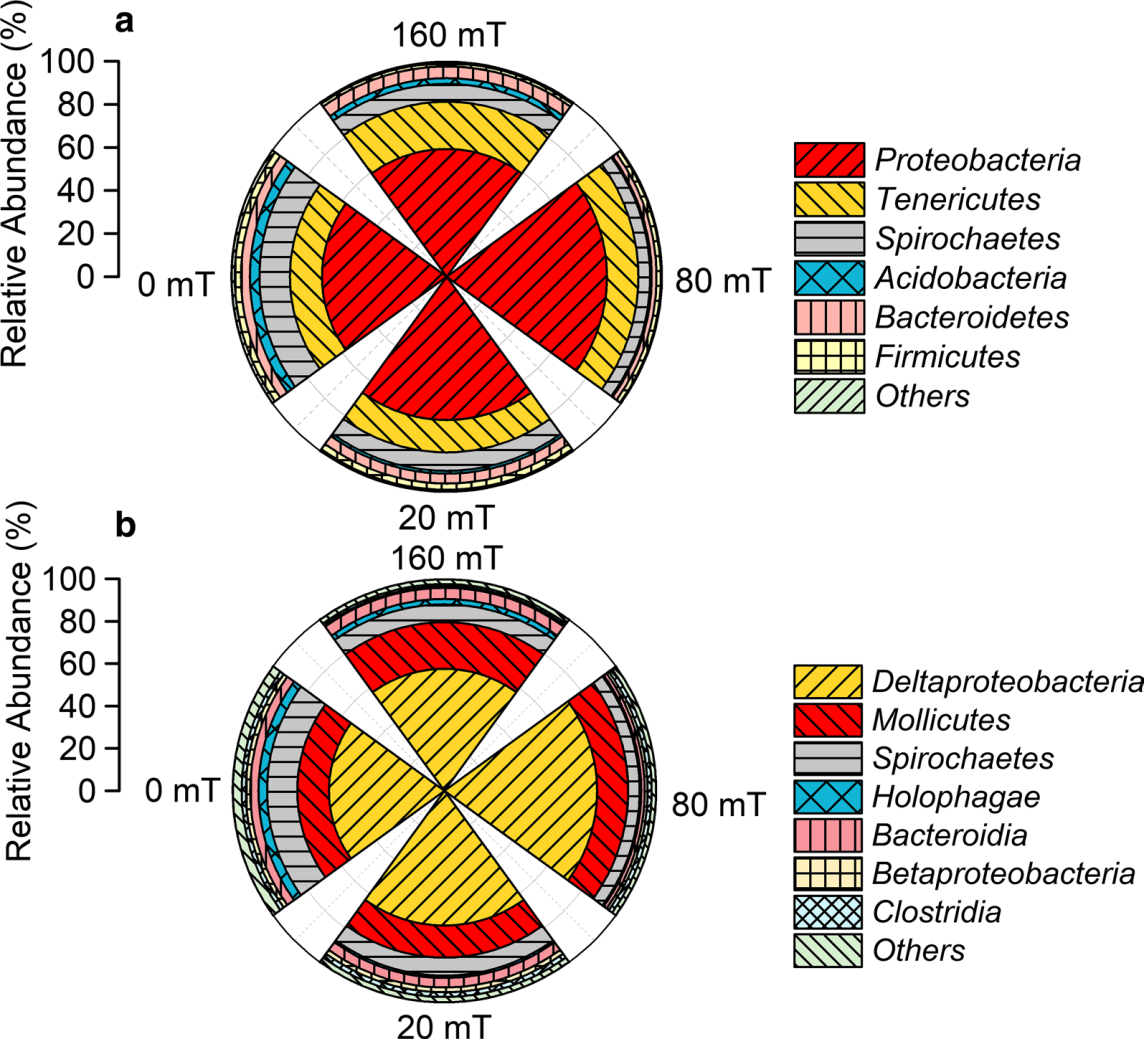
Relative abundance of predominant phyla (**a**) and classes (**b**) of the anode biofilms of MFCs. Phyla and classes that represent < 1% of the total bacterial community composition are classified as “others”

**Fig. 7:**
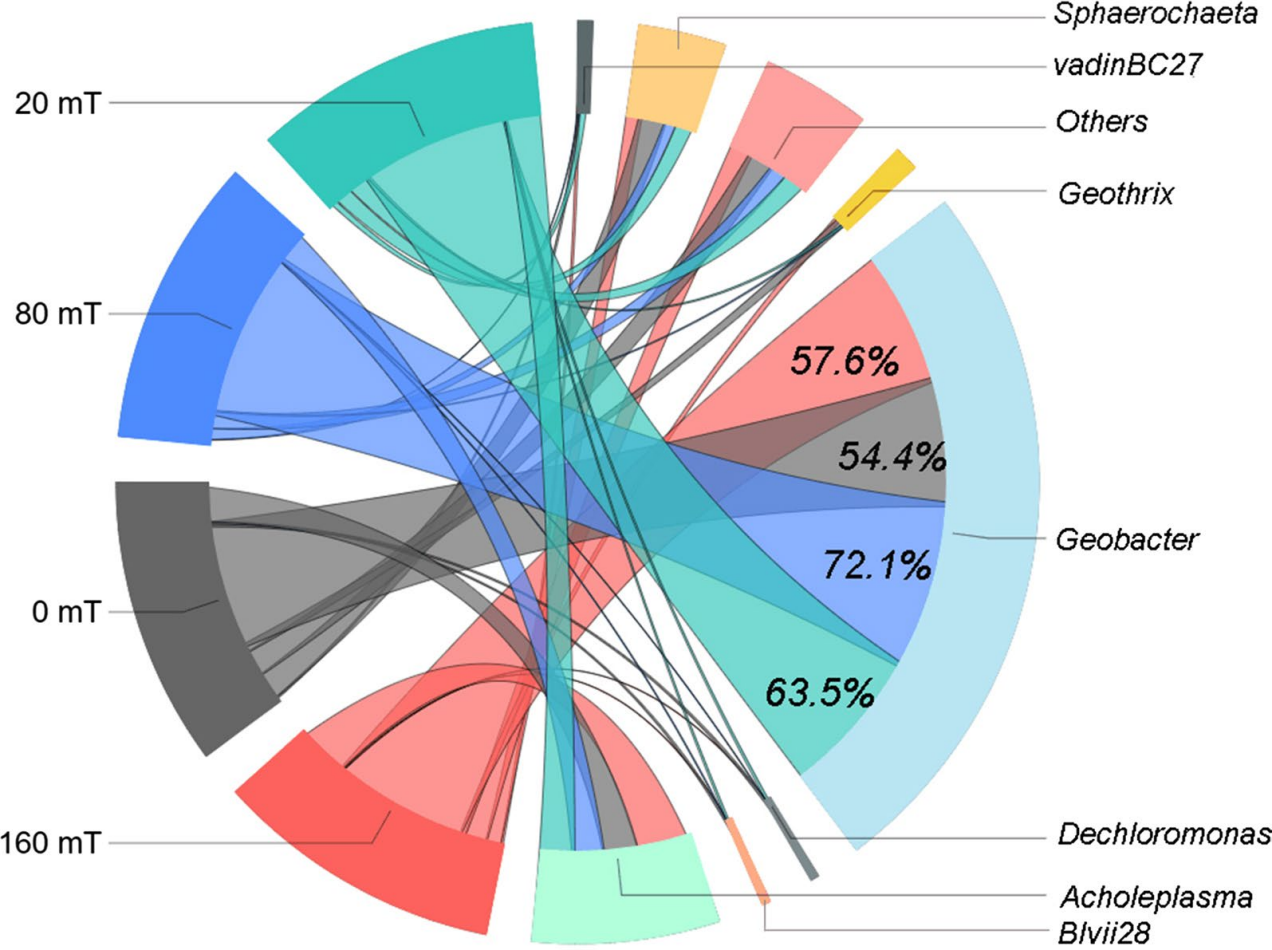
Taxonomic classification of bacterial 16S rRNA sequences in the anode biofilms of the MFCs at the genus level

At the phylum level, six bacterial phyla were identified with over 1.0% abundance and the dominating phyla were *Proteobacteria*, *Tenericutes*, and *Spirochetes* in each community (Fig. 6a). *Proteobacteria* as the most dominant phyla, accounted for over 50% of the bacteria in all of all the MFCs. The relative amount of *Proteobacteria* and *Tenericutes* was higher in magnetic MFCs than non-magnetic MFC, of which MFC-80 mT had the highest proportion of *Proteobacteria*, with abundances of 59.3%, 74.5%, 66.5%, and 57.8% for MFC-160 mT, MFC-80 mT, MFC-20 mT, and MFC-0 mT, respectively. However, the relative abundance of *Spirochetes* in magnetic MFCs (8.3%, 5.5%, and 8.6%, respectively) were lower than that in the non-magnetic MFC (14.2%). At the class level, the relative abundance of *Deltaproteobacteria* was higher in the magnetic MFCs, especially in MFC-80 mT, while the relative abundance of *Spirochetes* and *Holophagae* were highest in the non-magnetic MFC-0 mT (Fig. 6b). Further analysis at the genus level demonstrated that the predominant population in all of the MFCs belonged to the *Geobacter* (Fig. 7), which is a common exoelectrogens. The relative abundance of the *Geobacter* was highest in MFC-80 mT, followed by MFC-20 mT, MFC-160 mT, and MFC-0 mT, indicating that the SMF promoted the enrichment of exoelectrogens on the anode, thus improving the performance of the MFCs. The relative abundance of *Geobacter* in the magnetic MFCs (72.1%) was up to 32.5% higher than that of the non-magnetic MFC (54.4%) (Fig. 7). The increment of *Geobacter* under a static magnetic field was higher than under pulse magnetic field (6.6%, data in previous study) [27], suggesting that static magnetic field is more favorable for the enrichment of exoelectrogens. The appropriate magnetic field intensity for enriching exoelectrogens appears to be around 80 mT. Additionally, the non-magnetic MFC specially enriched for *Sphaerochaeta* (11.7%) compared to the magnetic MFCs, which has been observed in hydrogen-producing systems [38].

### Effects of the magnetic field on archaeal community structures of anode biofilms

**Fig. 8:**
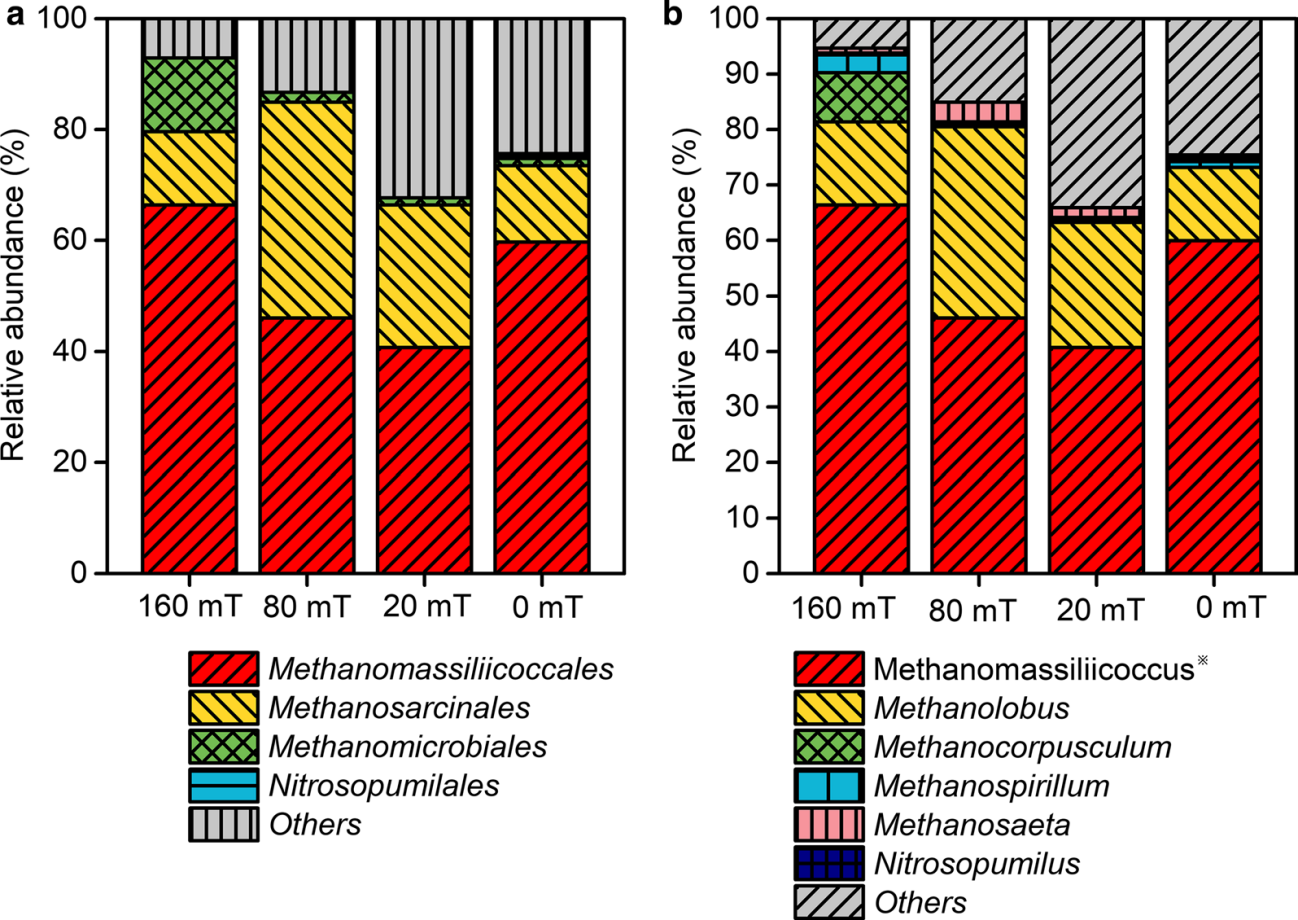
Taxonomic classification of archaeal 16S rRNA sequences in anode biofilms of the MFCs at the order level (**a**) and the genus level **b**. *Represents *Methanomassiliicoccus* with a similarity rate of 90% compared with *Methanomassiliicoccus luminyensis* strain B10 as based on BLAST analysis utilizing the National Center for Biotechnology Information (NCBI) database

*Methanomassiliicoccales* and *Methanosarcinales* accounted for 66–85% of the total sequences in each sample at the order level (Fig. 8). At the genus level, the predominant genera were affiliated with *Methanomassiliicoccus* with a similarity rate of 90% compared with *Methanomassiliicoccus luminyensis* strain B10 via BLAST analysis utilizing the National Center for Biotechnology Information (NCBI) database. The relative abundance values of *Methanomassiliicoccus* in MFC-160 mT, MFC-80 mT, MFC-20 mT, and MFC-0 mT were 66.4%, 46.0%, 40.7%, and 59.9%, respectively. It has been reported that *Methanomassiliicoccus* can use hydrogen as electron donor to produce methane [39]. As mentioned in bacterial community structures, *Sphaerochaeta* with higher relative abundance was enriched in the non-magnetic MFCs compared with the magnetic MFCs, while *Sphaerochaeta* can oxidize acetate to produce hydrogen gas that is further used by *Methanomassiliicoccus*. Since this process consumes acetate, it is not conducive to the enrichment of *Geobacter*. As such, we predicted that the lower amounts of *Sphaerochaeta* and *Methanomassiliicoccus* in the magnetic MFCs would result in the higher power generation compared with the non-magnetic MFC. The relative abundance of *Methanolobus* was highest in MFC-80 mT (34.5%), followed by MFC-20 mT (22.6%), MFC-160 mT (15.0%), and MFC-0 mT (13.3%). Growth and methanogenesis of *Methanolobus* are not supported with acetate or H_2_/CO_2_, but with methanol, monomethylamine, etc. [40], suggesting that there is no competition between exoelectrogens and *Methanolobus*.

## Conclusions

This work is the first to report the application of magnets as electrode materials in bioelectrochemical systems. Magnetic MFCs obtained much higher power generation and COD removal rate compared with the non-magnetic MFC. The SMF decreased the internal resistance of MFCs, especially in regard to its diffusion resistance. Illumina sequencing of 16S rRNA gene amplicons suggested that SMF promoted the enrichment of exoelectrogen (*Geobacter*), and the optimal MF intensity was around 80 mT. This study provides a magnetic method for exoelectrogen enrichment, which can be extensively applied in bioelectrochemical systems. The further investigation on low resistance magnetic electrode and magnetic material with high conductivity is important to construct magnetic BESs with low internal resistance. Furthermore, to reveal the mechanism of EET stimulation by magnetic field, the molecular regulation of EET-related genes and proteins needs to be investigated using multi-omic tool in the future.

## Data Availability

The datasets used and/or analyzed during the current study are available from the corresponding author on reasonable request.

## Abbreviations

MFCs: microbial fuel cells
SMF: static magnetic field
MFs: magnetic fields
BESs: bioelectrochemical systems
EET: extracellular electron transfer
